# Genomic modulators of gene expression in human neutrophils

**DOI:** 10.1038/ncomms8545

**Published:** 2015-07-07

**Authors:** Vivek Naranbhai, Benjamin P. Fairfax, Seiko Makino, Peter Humburg, Daniel Wong, Esther Ng, Adrian V. S. Hill, Julian C. Knight

**Affiliations:** 1 Wellcome Trust Centre for Human Genetics, Nuffield Department of Medicine, University of Oxford, Oxford OX3 7BN, UK.

## Abstract

Neutrophils form the most abundant leukocyte subset and are central to many disease processes. Technical challenges in transcriptomic profiling have prohibited genomic approaches to date. Here we map expression quantitative trait loci (eQTL) in peripheral blood CD16+ neutrophils from 101 healthy European adults. We identify *cis*-eQTL for 3281 neutrophil-expressed genes including many implicated in neutrophil function, with 450 of these not previously observed in myeloid or lymphoid cells. Paired comparison with monocyte eQTL demonstrates nuanced conditioning of genetic regulation of gene expression by cellular context, which relates to cell-type-specific DNA methylation and histone modifications. Neutrophil eQTL are markedly enriched for trait-associated variants particularly autoimmune, allergy and infectious disease. We further demonstrate how eQTL in *PADI4* and *NOD2* delineate risk variant function in rheumatoid arthritis, leprosy and Crohn’s disease. Taken together, these data help advance understanding of the genetics of gene expression, neutrophil biology and immune-related diseases.

## Abstract (web summary)

Neutrophils are the most abundant subset of leukocyte and central to many diseases. Here by mapping expression quantitative trait loci (eQTL) in the context of epigenetic marks in neutrophils and monocytes of 101 healthy European adults, the authors provide a resource to advance understanding of immune-related trait-associated genetic variants.

## Graphical abstract

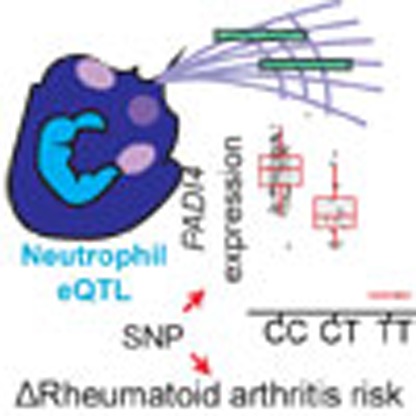

Variation in the human genome is a major regulatory mechanism of gene transcription^1^. Regulatory variants may modulate gene expression of local genes (*cis*-eQTL, likely acting on the same chromosome) or genes at a distance on non-contiguous chromosomes (*trans*-eQTL). Quantitative variation in transcription frequently leads to protein variation^2^ leading to phenotypic traits. Although cell-type-specific effects were noted in early studies^3^, ease of sample availability may explain why the largest studies of eQTL are in cell lines^4^,^5^,^6^,^7^,^8^ or mixed populations of leukocytes from peripheral blood^9^,^10^,^11^,^12^. Studies of eQTL in monocytes^13^,^14^,^15^, monocyte-derived dendritic cells^16^, B lymphocytes^13^, regulatory T cells^17^, CD4+ T cells or bulk T cells^3^ demonstrate that the effects of a genetic variant on gene expression differ according to cell type and these are further conditioned by cellular activation state and stimulatory environment^18^,^19^,^20^. Similar results comparing tissues support this conclusion^21^,^22^,^23^,^24^. Collectively, these studies demonstrate the need for context-specific eQTL mapping in diverse primary populations of cells informative for disease.

Neutrophils make up 40–70% of the total circulating leukocyte pool and due to their abundance in blood and tissue, they are frequently observed in tissue specimens with minimal blood contamination. There has been one recent study of eQTL in murine neutrophils^25^, but the genetic architecture of gene expression in human neutrophils remains unclear. Challenges in isolating neutrophils^26^ (as opposed bulk granulocytes that paradoxically bias RNA measures towards eosinophils^27^) have limited the ability to study these cells leading to attempts at *in silico* prediction of neutrophil eQTL^28^ from whole-blood data. About 10^11^ neutrophils are produced daily in the adult human bone marrow and constitute first-responders in the innate immune response to a variety of infectious and non-infectious insults^29^. Neutrophils are characterized by multilobed nuclei and an abundance of primary, secondary and tertiary soluble-defence-mediator-filled granules. Although neutrophils are classically thought to be short-lived cells, they play roles in acute and chronic inflammation and can migrate into tissues or return to the blood compartment and survive^30^. Neutrophils have extensive crosstalk with each of the major blood cell subsets (megakaryocyte, myeloid and lymphoid)^31^,^32^,^33^,^34^ further magnifying their function in health and disease. Neutrophils are thus central to orchestrating immune responses and understanding the regulation of gene expression in neutrophils may, we hypothesized, offer insights into disease biology.

We identified *cis*-acting genetic modulators of gene expression in neutrophils for more than 3,000 genes, including dozens involved in the development, migration and function of neutrophils. Comparison with other cell types and paired analysis of neutrophil and monocyte eQTL demonstrates how cell-type modifies the effect of eQTL. Moreover, cell-type-specific epigenetic data help resolve the mechanisms of cell-type constraint of eQTL and fine-map causal variants. We show how variants that affect gene expression are implicated in hundreds of common diseases. We interrogate two such associations. First, we show how integration of neutrophil and monocyte eQTL and epigenetic data with genome-wide association study (GWAS) data implicates PADI4 expression in neutrophils in rheumatoid arthritis susceptibility. Second, we show how a single-nucleotide polymorphism (SNP) with pleiotropic association with leprosy and Crohn’s disease (CD) susceptibility alters neutrophil inflammatory responses to NOD2 ligands through altered STAT3 binding and consequent NOD2 expression. Finally, we observe that many neutrophil eQTL reside within regions that have been subject to selection. Collectively, our data advance the understanding of the genetics of gene expression for a pathophysiologically important cell type and role in human disease.

## Results

### Defining eQTL in primary human neutrophils

**Figure 1:**
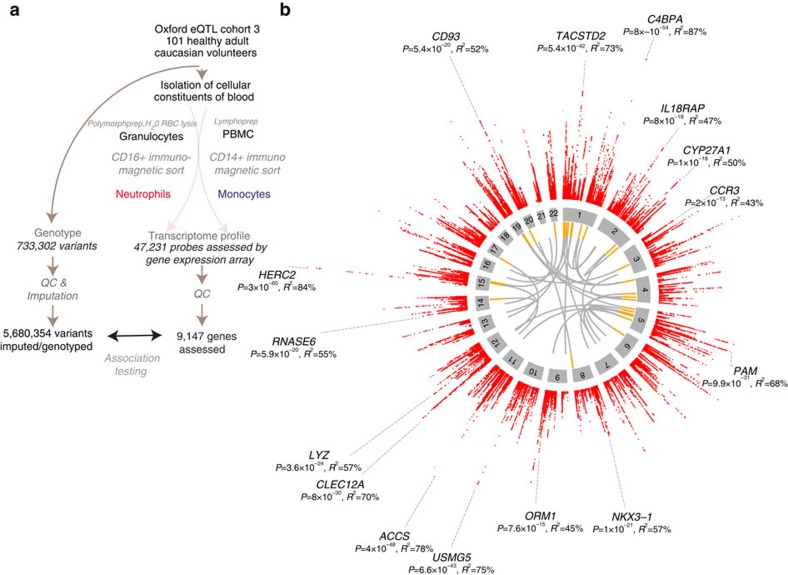
Study schema and overview of eQTL in primary human neutrophils. (**a**) Flowchart showing the schema of this study to identify eQTL in human neutrophils from healthy adult volunteers of European ancestry with genotypes determined by array-genotyping and by imputation tested for association with global gene expression in *cis* (variant <1 Mb of gene) or *trans* (variant on non-contiguous chromosome to gene). This analysis was contemporaneous to our previously reported study^19^ of monocytes enabling direct comparison of genetic correlates of gene expression between neutrophils and monocytes. (**b**) Circos plots for the neutrophil dataset. Outermost rim (red dots) shows a Manhattan plot for significant *cis*-eQTL (FDR<0.05) with names of genes with large effect sizes (variance explained, *R*^2^>45%) or lowest five *P*-values; the second rim (grey boxes) shows the chromosome ideogram with chromosome number within each box; the third rim (orange boxes) indicates a gene to which a significant (FDR<0.01) trans-eQTL was observed; and the innermost spokes (grey) connect trans-acting variants to the gene the variant is associated with.

To identify regulatory variants for gene expression in primary human neutrophils, we enrolled 101 healthy adult European volunteers in Oxford, UK (Fig. 1a). Individuals were genotyped on the Human OmniExpress 12v1.0 chip, and we used genome-wide imputation with stringent quality checks to infer additional SNP or simple insertion/deletion genotypes with high confidence. CD16+ neutrophils, isolated by a two-step sequential gradient-density and immunomagnetic sorting procedure yielding a highly purified population, were subjected to whole-transcriptome characterization by hybridization of cRNA to Human HT-12 v4 Expression BeadChips (Illumina). We assessed only genes for which specific probes existed, and which were confidently detected in >5% of the cohort (GenomeStudio detection *P*<0.01). We used a widely adopted linear-additive modelling approach (MatrixeQTL)^35^ adjusting for principal components which has been shown to enhance eQTL discovery and control for technical and demographic heterogeneity^10^,^19^,^28^,^36^.

After genome-wide imputation 3,281 genes (roughly (∼) 30% of 9,147 genes tested and equivalent to 3,675 probes) had one or more identifiable loci within 1 Mb (defined as *cis*-eQTL) of a probe that was significantly associated with gene expression at a false discovery rate (FDR) threshold of 5% (Fig. 1b, Supplementary Data 1). The median proportion of variance explained by the most significant variant for each probe, was 16.6% (interquartile range (IQR) 12.9–26.0%) but notably was >50% for several genes, such as *C4BPA*, involved in venous thrombosis^37^, the anti-inflammatory monosodium urate receptor *CLEC12A*^38^ and the antibacterial enzyme lysozyme (*LYZ*; Additional genes with large effect sizes are highlighted in Fig. 1b). Genes with an eQTL were more highly expressed (median normalized expression 8.14 versus 7.7, *P*=1.7 × 10^−72^) and had greater variance (median variance 0.049 versus 0.029, *P*=1.07 × 10^−122^) than genes without an eQTL. The most significant eQTL per gene (denoted as the peak eQTL) clustered around the transcriptional start site (TSS), and the effect sizes increased with proximity to the TSS (Supplementary Fig. 1a) but, annotation and inspection at higher resolution demonstrates distribution of eQTL across gene structures with clustering around TSS and transcription end sites, consistent with previous studies^39^ (Supplementary Fig. 1b).

We did not identify association between any of 168 copy-number variants, inferred from raw genotype calls, and gene expression after correcting for multiple comparisons (Supplementary Data 2).

Comprehensive identification of loci associated with gene expression on non-contiguous chromosomes (*trans* eQTL) typically requires large sample^12^ sizes due to typically reduced effect sizes of variants acting *trans* allied to an increased burden of multiple hypothesis testing. Accordingly, we identified a smaller set of 33 genes regulated in *trans*, at a false discovery rate threshold of <1% (Fig. 1b, Supplementary Data 3). These *trans* eQTL include rs10012416, which we find is a *cis*-eQTL for *CRIPAK* (encoding an inhibitor of p21 activated kinase Pak1 important in neutrophil cytoskeletal dynamics involved in phagocytosis^40^) and has *trans* effects on *AVP*, *SPTBN3* and *IRF6*; and rs10784774, a *cis*-eQTL for *LYZ* that is associated with *ZNF131* in neutrophils as we previously had reported in monocytes^13^. We note that this study is not powered to identify trans eQTL with weak to moderate-sized effects.

### eQTL in genes central to neutrophil biology

**Figure 2:**
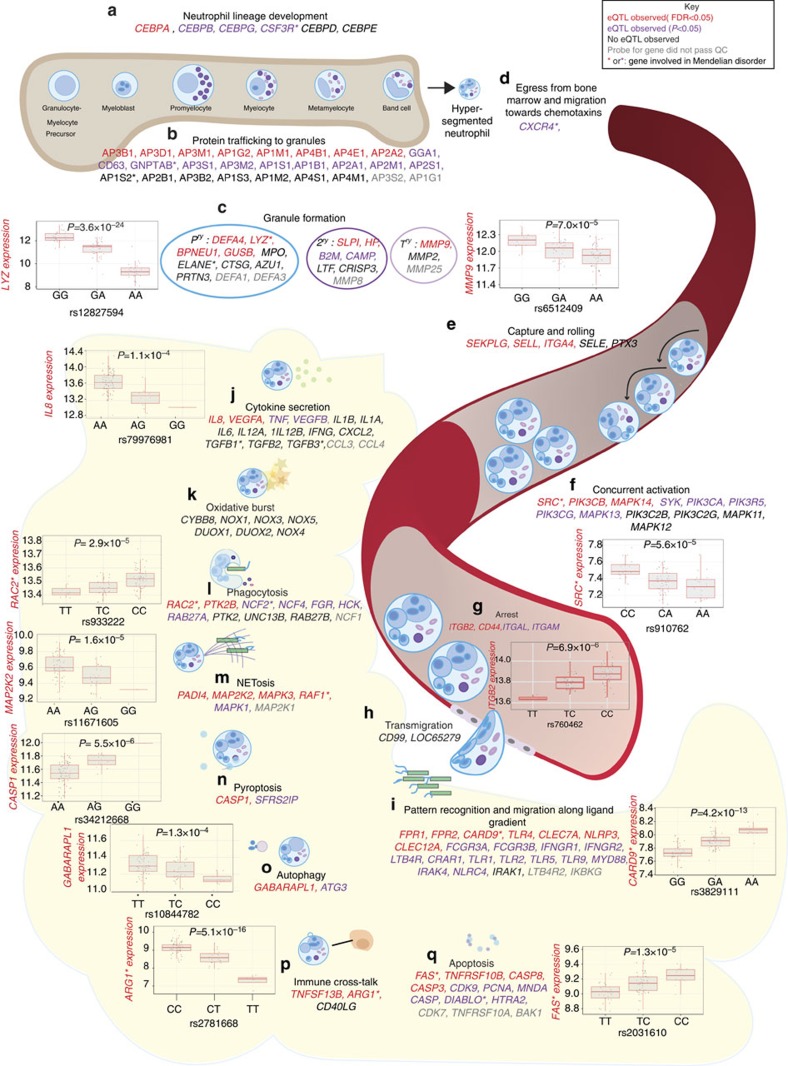
eQTL in genes involved in neutrophil biology. As a narrative resource, we compiled a list of 164 genes of importance in neutrophil biology^29^,^30^,^41^,^42^,^43^ and illustrate their role together with eQTL information (denoted be gene colour). Probes for 113 genes passed QC: 9 genes had no eQTL (black gene names) and 104 had an eQTL (47 with FDR<0.05 red gene names, 57 with *P*<0.05 but FDR>0.05 purple gene names). Genes involved in nearly every aspect of neutrophil biology have identifiable eQTL and several are involved in Mendelian disorders (asterisked genes) for example *RAC2* (neutrophil immunodeficiency syndrome) and *FAS* (autoimmune lymphoproliferative syndrome type 1A). (**a**) Granulocyte–monocyte progenitor cells (GMP) respond to GM-CSF stimulation of a receptor encoded by *CSFR3* to form neutrophils under the instruction of CEBPα and other CEBP transcription factors. Neutrophil development through various stages is coincidental with expression of proteins that traffic to primary, secondary and tertiary granules. (**b**) AP heterotetramers are involved in trafficking of proteins such as LYZ (involved in renal amyloidosis) and MMP9 (involved in metaphyseal osteolysis, nodulosis and arthropathy) to (**c**) primary, secondary and tertiary granules. (**d**) Hypersegmented neutrophils egress from the bone marrow in response to IL-8 and SDF-1, and are (**e**) captured on endothelium within blood vessels through interaction of selectins and ligands such as SEKPLG and SELL. (**f**) Capture, rolling and eventual arrest on endothelial surfaces is associated with activation, mediated in part by PI-3 and MAPK pathways. (**g**) Arrest of neutrophils is mediated by α1β2/αMβ2 integrin heterodimers encoded by *ITGAL*, *ITGBA2*, *ITGAM* and (**h**) rapid para- and *trans*-cellular migration ensues. (**i**) Neutrophils express pattern recognition receptors and receptors for host derived molecules allowing detection of bacteria and effective migration. In tissues, neutrophil response includes (**j**) cytokine secretion, (**k**) oxidative burst, (**l**) phagocytosis, (**m**) production of neutrophil extracellular traps (NETosis), (**n**) pyroptosis, (**o**) autophagy, (**p**) crosstalk with other immune, and (**q**) eventual cell death via apoptosis should cell death not have eventuated from another response. Box lower and upper border denote 25th and 75th centiles, respectively, central line denotes median and whiskers extend to 1.5 × IQR. In all cases 101 donor replicates shown.

We proceeded to examine genes relevant to neutrophil function for eQTL as these may serve as a narrative resource to understanding the role of regulatory variants in neutrophil biology. We assembled a list of 164 genes described, with a verifiable reference, to be involved in a particular aspect of neutrophil function as being involved in aspects of neutrophil function in recent reviews^29^,^30^,^41^,^42^,^43^. Because this approach is at risk of narrative bias, we caution against interpretation of these results as evidence of enrichment for genes important in neutrophil function. Of the 113 genes for which an expression probe existed, passed QC and was included in analysis, 104 had an identifiable variant associated with expression (*P*<0.05) and 47 after false-discovery adjustment (FDR<0.05). We observe eQTL for genes involved in most aspects of neutrophil development and function (Fig. 2, Supplementary Data 4). Interestingly, several genes with an eQTL are implicated in Mendelian disorders involving neutrophils. For example rs933222 is associated with expression of *RAC2*, a gene encoding a Rho GTPase that is part of the NADPH oxidase complex involved in initiation of phagocytosis (Fig. 1l) and is involved in neutrophil immunodeficiency syndrome^44^ (OMIM #608203).

Ingenuity pathway analysis of 975 genes with an eQTL in neutrophils but not in monocytes (Supplementary Fig. 2, and detailed further below), revealed enrichment for functions relating to cell death (*P*=1.4 × 10^−7^), apoptosis (8.2 × 10^−7^), necrosis (6.4 × 10^−6^); and infection, notably viral infection (4.2 × 10^−6^) and infection of cells (2.2 × 10^−5^). The most significant upstream transcriptional regulator was TP53, which plays a critical role in cell proliferation and apoptosis as well as antimicrobial function highlighting how the consequences of TP53 for the individual may be modulated by eQTL in downstream mediator and effector genes. A number of cytokines were also identified as upstream regulators including IFNB1 (*P*=5.9 × 10^−4^), IL15 (3.0 × 10^−3^), IFNG (4.1 × 10^−3^), CD40LG (6.8 × 10^−3^) and TNF (7.2 × 10^−3^).

### Patterns of eQTL in neutrophils and other immune cell types

Regulation of gene expression may be constrained to specific cell types and contexts. To delineate aspects of shared and unique regulatory genomic architecture in neutrophils, we pursued three complementary approaches.

First, we note that 63% (2069/3281) of genes with a *cis*-eQTL in neutrophils are reported to have a *cis*-eQTL in the largest blood eQTL meta-analysis to date^12^ (obtained through the bloodeqtl browser) and recent *in silico* predictions^28^ attribute 20% (443/2188) to neutrophils and 90% (1969/2188) as ‘generic’ (note that a gene may be denoted as neutrophil *and* generic). Therefore, many eQTL in neutrophils may not have been identified in whole-blood studies, and even when they are, *in silico* deconvolution of cell types may not establish if a gene has an eQTL in a given cell type. Conversely, 84% of genes (415/495) with an eQTL in blood that are bioinformatically ascribed to neutrophils (and tested in our study) have an eQTL in purified neutrophils, providing evidence of cross-study validation. The estimated effect sizes in neutrophils and whole blood for genes with an eQTL in both are only moderately correlated (spearman correlation estimate for 691 genes, rho=0.54, 95%CI 0.43–0.60, *P*=1.77 × 10^−23^); as we shall present later, directionally opposing eQTL amongst different cells are not uncommon and this may plausibly affect *in silico* effect size estimates. Second, for 40% (261/646) of genes with a *cis*-eQTL in mouse CD4+ T cells and/or neutrophils^25^ whose human homologues we tested in humans, we observe a significant *cis*-eQTL in neutrophils (a 5.72-fold enrichment over random expectation (646/9147), *P*=1.02 × 10^−80^). Third, we compared genes with an eQTL in neutrophils to those that have been reported previously in other primary immune cells: CD4 T-cells, regulatory T cells^17^ and B cells^13^ (grouped as lymphoid cells) or monocytes and monocyte-derived dendritic cells^14^,^15^,^16^,^19^ (grouped as myeloid cells). Of the 3281 genes with an eQTL in neutrophils, 1,671 (51%) had an eQTL in both myeloid and lymphoid cells, 823 (25%) in non-neutrophil myeloid cells, 337(10%) in lymphoid cells and 450 have no reported eQTL in either myeloid or lymphoid cells (Supplementary Fig. 3). Although our analysis neither assesses whether the same genetic variant regulates gene expression in all cell types nor whether the effect is the same, it demonstrates that, for at least 14% of the genes in which we observe an eQTL, we have identified novel regulatory variants and shown the utility of eQTL mapping in primary neutrophils.

**Figure 3:**
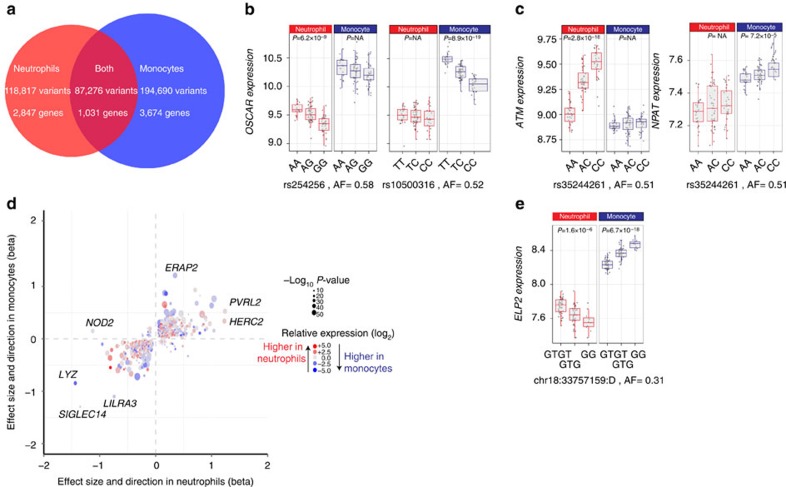
Shared and cell-type-specific *cis*-eQTL in neutrophils and monocytes. (**a**) Venn diagrams showing the number of variants and genes with *cis*-sQTL by cell type. Examples of cell-type constraint include (**b**) independent variants associated with expression of the same gene, *OSCAR*, specific to either neutrophils or monocytes (lead eSNPs rs254256 and rs1050031, respectively, *r*^2^<0.02). Conversely, (**c**) a single variant may be associated with different genes in each cell type as shown for rs35244261 (associated with elevated expression of *ATM* in neutrophils, and *NPAT* in monocytes). (**d**) Amongst 1,939 genes that have an eQTL in both cell types, 1,031 involve the same variant and are plotted with the effect size in neutrophils and monocytes (shown on *x*- and *y* axes, respectively). Colour denotes the relative expression in neutrophils and monocytes, and size denotes the minimum *P*-value of an eQTL for that gene. Several eQTL show divergent direction of effects on the gene they regulate including *NOD2*, *THBD*, *TSTD1*, *TOM1L2* and *ELP2* (latter shown in **e**). Genes with large effect sizes are highlighted, including *HERC2*, the gene responsible for iris colour, which has amongst the most significant eQTL of all genes in neutrophils. *P*-values >0.05 are denoted as *P*=NA. Box lower and upper border denote 25th and 75th centiles, respectively, central line denotes median and whiskers extend to 1.5 × IQR. In all cases 101 donor replicates shown.

To further examine the effect of cell type on eQTL, we performed a detailed analysis of monocytes and neutrophils isolated contemporaneously from 99 caucasian donors in which we confined analysis to 8,362 genes expressed in both cell types. We identified 400,783 unique variant-gene associations across both cell types: 87,276 variants for 1031 genes in both neutrophils and monocytes, 118,817 variants for 2,847 genes in neutrophils and 194,690 variants for 3,674 genes in monocytes (Fig. 3a). Higher expression of a gene in neutrophils compared with monocytes was a positive predictor of whether an eQTL was present in neutrophils or not, but normalized probe intensity explains just ∼2% of variance and there are many genes in which an eQTL is observed in only one of neutrophils or monocytes despite similar levels of gene expression. The effect size of regulatory variants in both monocytes and neutrophils was larger for eQTL seen in both cell types compared with those seen only in one cell type (*P*=7.4 × 10^−196^, Supplementary Fig. 4) confirming previous observations^13^. Interestingly, whereas the number of unique eQTL is greater in monocytes, eQTL effect sizes were larger in neutrophils than in monocytes regardless of whether the eQTL was seen in both cell types (*P*=1.9 × 10^−19^) or just one (*P*=9 × 10^−200^). This likely reflects a proportionally greater impact of genetic variation in governing gene expression in neutrophils that recapitulates their shorter lifespan and reduced exposure to environmental modifiers.

We highlight three patterns of cell environment-modifying genetic effects on gene expression in addition to apparent cell-type constraint of regulatory activity. First, although 1,939 genes have at least one eQTL in both cell types (and 1,031 share an eQTL), for 908 genes the peak regulatory variants are different and for 840 (93%) of these, independent (*r*^2^<0.2) demonstrating that the same gene may have independent regulatory variants of varying strengths and directions in different cell types. An example for the *OSCAR* gene is shown in Fig. 3b. Conversely a particular variant may show pleiotropy in the gene it regulates in neutrophils and monocytes. About 4.5% (12,661/278,200) of variants that regulate a gene in one cell-type are associated to a different gene in the other cell type. For example, rs35244261 is associated with levels of *ATM* in neutrophils and *NPAT* in monocytes (Fig. 3c). In gene-dense regions this could be due to cell-type conditioning the effect of one or more variants that have regulatory effects on nearby genes. The third pattern we highlight is pleiotropy in direction of effect of a variant in different cell types on the same gene. We identified 2,823 variants with significant but directionally opposing effects on 66 genes in both cell types (Fig. 3d). Several are notable for the variant being associated with a disease directly or through a linked variant such as rs8066560 (*TOM1L2*, Parkinson’s disease), chr18:3375159:D (Fig. 3e, *ELP2*, oesophageal squamous cell carcinoma), rs74058715 (*PADI4*, rheumatoid arthritis), and rs1981760 (*NOD2*, leprosy and CD), the latter two described in detail below. Collectively, the comparison of monocyte and neutrophil eQTL supports a model of widespread interaction between cellular milieu and genetic factors in regulation of gene expression even amongst cells of similar (myeloid) lineage. Moreover, delineation of cell-type restriction of eQTL in neutrophils may provide a tool to understand their involvement in variant-phenotype association.

### Epigenetic mechanisms of eQTL in neutrophils and monocytes

**Figure 4:**
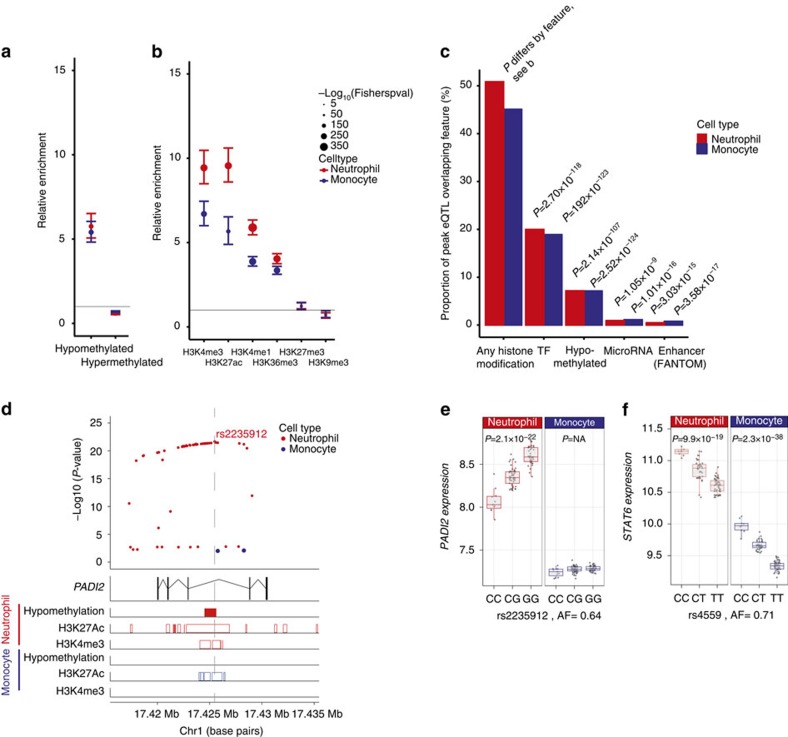
Epigenetic basis of eQTL in neutrophils and monocytes. We integrated eQTL data with whole-genome bisulfite sequencing and chromatin-immunoprecipitation sequencing data from primary neutrophils and monocytes from 4 to 8 individuals from the BLUEPRINT Consortium. (**a**) Enrichment of *cis*-eQTL in neutrophils and monocytes, in hypo- and hypermethylated regions of the genome in concordant cell types relative to all SNP’s tested for eQTL. (**b**) Enrichment of neutrophil and monocyte *cis*-eQTL relative to all variants tested for colocalisation in regions of the genome associated with modified histones based on ChIP-Seq of modified histones in neutrophils and monocytes. (**c**) Proportion of *cis*-eQTL in neutrophils (*n*=3,774) and monocytes (*n*=4,668) that overlie modified histones in the concordant cell type (BLUEPRINT), transcription factor binding sites in cell lines (ENCODE), sites that are hypomethylated in the concordant cell type (BLUEPRINT), target sites of conserved miRNAs with high mirSVR scores (microRNA.org) or CAGE-seq defined enhancer sites in neutrophils and monocytes (FANTOM5). Note that an eQTL may overlap more than one feature. (**d**,**e**) An example of a cell-type-specific eQTL possibly attributable to methylation differences in cell types is rs2235912, a site which is in an intron of *PADI2* that is hypomethylated in neutrophils but not monocytes. This locus is marked by the activating histone marks H3K27Ac and H3K4me3 in neutrophils but in monocytes has a smaller region marked by H3K27Ac only. As shown in **e**, the gene is more highly expressed in neutrophils than monocytes, perhaps due to the greater activating histone marks in neutrophils. The G allele creates an additional putative CpG site, which may explain the allele being associated with higher *PADI2* expression relative to the C allele. (**f**) STAT6 is an example of a gene with an eQTL in both monocytes and neutrophils where the eQTL lies in a predicted binding region for mir-18b, a microRNA. Tails in **a**,**b** show 95% CI of the enrichment estimate. In **e**,**f**, box lower and upper border denote 25th and 75th centiles, respectively, central line denotes median and whiskers extend to 1.5 × IQR. In all cases 101 donor replicates shown. *P*-values >0.05 are denoted as *P*=NA.

The local genomic environment of a regulatory variant is cell-type dependent and this may modify variant effect. Moreover, as has been shown for complex traits, leveraging epigenetic mark information may help fine-map genotype-phenotype associations^45^. Therefore, to elucidate the role DNA methylation and histone modification play in genetic effects on gene expression, we compared eQTL maps in neutrophils and monocytes to maps of DNA methylation and several histone modifications generated by whole-genome bisulfite sequencing or ChIP and sequencing (ChIP-Seq), respectively, in neutrophils and monocytes from 4 to 8 individuals by the BLUEPRINT consortium^46^. As may be predicted by their shared ontology, monocytes and neutrophils have substantial overlap in regions of the genome that are methylated or subject to histone modifications, although this differs by the specific combination of epigenetic mark (Supplementary Data 5). Relative to all imputed variants within 1 MB of a gene (4,812,,340 variants near a neutrophil-expressed gene tested for being a cis-eQTL), we observed marked and significant enrichment of peak *cis*-eQTL in hypomethylated regions (*P*_neut_=2.14 × 10^−107^, *P*_mono_=2.52 × 10^−124^) and depletion in hypermethylated regions (*P*_neut_=6.36 × 10^−46^, *P*_mono=_7.55 × 10^−23^) in the concordant cell type that is, neutrophil eQTL in neutrophil hypomethylated regions (Fig. 4a,c, Supplementary Data 6). Similarly, as shown in Fig. 4b (and Supplementary Data 6), peak *cis*-eQTL were enriched in regions with histone marks associated with promoter activity (H3K4me3; *P*_neut_=8.23 × 10^−231^, *P*_mono_=1.31 × 10^−165^), active or poised enhancers (H3K27Ac; *P*_neut_=4.85 × 10^−230^, *P*_mono_=1.34 × 10^−80^, H3K4me1; *P*_neut=_2.23 × 10^−308^, *P*_mono_=4.84 × 10^−229^) or activation (H3K36me3; *P*_neut_=2.01 × 10^−238^, *P*_mono_=3.22 × 10^−196^) and depleted in regions associated with repressive histone modifications (H3K27me3; *P*_neut_=3.78 × 10^−3^, *P*_mono_=2.59 × 10^−2^ and H3K9me3; *P*_neut_=3.35 × 10^−4^, *P*_mono_=2.57 × 10^−2^). These data are replicated by an orthogonal approach, in which, despite vastly fewer regions, we observe 14-fold enrichment of neutrophil eQTL in neutrophil enhancer regions (*P*=3.03 × 10^−15^) and 6-fold enrichment of monocyte eQTL in monocyte enhancer regions (*P*=3.58 × 10^−17^) using enhancer maps generated by CAGE-Seq in FANTOM5^47^,^48^. These data support a model of epigenetic environment-modifying regulatory effects in different cell types. An example for the *PADI2* gene is shown in Fig. 4d,e.

Mechanisms by which regulatory variants operate include alteration in transcription factor binding. Neutrophil and monocyte peak eQTL are 3.1- and 2.87-fold enriched (2.70 × 10^−118^ and 1.92 × 10^−123^, Supplementary Data 6), respectively, in regions bound by a transcription factor in cell lines studied in the ENCODE project^49^. Expression levels of 44% (415/943) of reported human transcription factors^50^ that are expressed in both cell types differ by >0.5 log_2_ between neutrophils and monocytes (Supplementary Data 7), making it plausible that alteration in transcription factor binding sites in addition to differences in transcription factor abundance could lead to eQTL being observed in one cell type and not the other. About 1% (52 in neutrophils, 35 in monocytes) of peak eQTL lie within microRNA binding sites, a 3.44- (*P*=1.05 × 10^−9^) and 4.14-fold (1.01 × 10^−16^) enrichment for neutrophils and monocyte respectively (Supplementary Data 6 and 8). For example, rs4559 is a common variant associated with *STAT6* expression (Fig. 4f) in both cell types and is within the binding region of miR-18b. We note that although this analysis does not take into account whether the microRNA is expressed in the cell-type, the results are consistent with some eQTL having their effect through microRNA-directed transcriptional gene silencing^51^.

### Neutrophil eQTL are enriched for disease-associated variants

**Figure 5:**
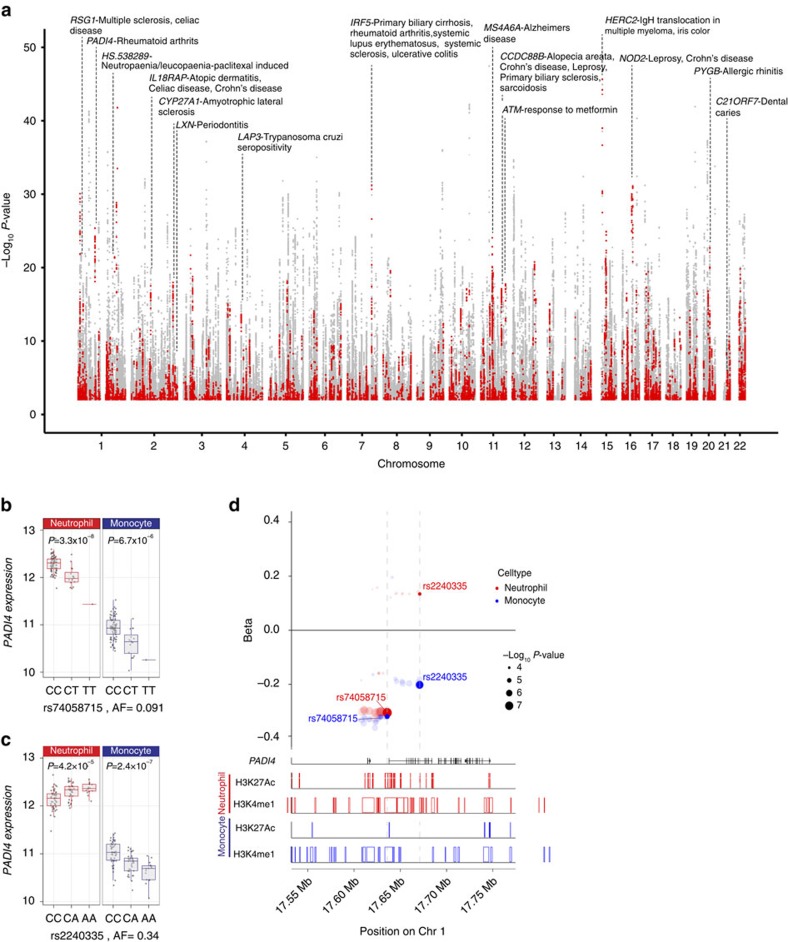
eQTL in neutrophils and their association with complex disease or trait. (**a**) Manhattan plot showing eQTL in neutrophils highlighting those associated with or in linkage disequilibrium (*r*^2^>0.8) with a disease/trait associated variant listed in the NHGRI GWAS catalog. Each point denotes a single eQTL and is coloured red if the locus is associated with a complex trait or grey if not. (**b**–**d**) An example in *PADI4* demonstrates how integrated analysis of an eQTL informs understanding of rheumatoid arthritis (RA) risk. Expression level of *PADI4* is associated with two independent variants, (**b**) rs2240335 and (**c**) rs74058715 (*r*^2^=0.02). rs2240335-A, the derived allele, is associated with elevated expression of *PADI4* in neutrophils and diminished levels in monocytes. rs7405871-T is associated with reduced *PADI4* expression in neutrophils and monocytes. rs2240335 is in near-complete linkage with rs2301888, and this locus is associated with RA risk^54^ (**d**) Compiled plot showing effect size estimate (beta) for variants associated with *PADI4* expression in neutrophils and monocytes (upper panel), relative to genic structures (track two), and BLUEPRINT ChIP-Seq reads for two histone marks in neutrophils (tracks three and four) and monocytes (tracks 5 and 6) for H3K27Ac and H3K4me1 demonstrating that rs2240335 lies in a region marked by both H3K27Ac and H3K4me1, a marker of active enhancers, in neutrophils but not in monocytes.In **b**,**d**, box lower and upper border denote 25th and 75th centiles, respectively, central line denotes median and whiskers extend to 1.5 × IQR. In all cases 101 donor replicates shown.

eQTL mapping can provide functional insight into the basis for genetic association of complex traits. We systematically examined eQTL in neutrophils for association with complex traits. Of all eQTL in neutrophils 7.5% (14,390/191,767) are associated with one or more of 327 traits (of 1,138 unique disease/traits curated in the NHGRI GWAS catalogue^52^) directly or through linkage (*r*^2^>0.8) with the trait-associated variant (Supplementary Data 9). This represents a 2.7-fold (95% CI 2.67–2.77, *P*<2.2 × 10^−16^) enrichment relative to all SNPs originally tested for association with gene expression (eQTL). Notable examples are highlighted in Fig. 5a. Reciprocally, 7.8% (14,390/183,124) of trait-associated variants are, or tag, eQTL in neutrophils.

Collation of GWAS traits into disease categories demonstrates that enrichment is observed for gastrointestinal disorders (7.2-fold enrichment; *P*<2 × 10^−308^), allergy (6.3-fold enrichment; *P*=1.3 × 10^−117^), autoimmune (6.3-fold enrichment, *P*<2 × 10^−308^) and infectious diseases (3.1-fold enrichment, *P*=7.9 × 10^−137^) grouped together or disaggregated for viral (3.7-fold enrichment, *P*=1.8 × 10^−56^), parasitic (5.1-fold enrichment, *P*=6.4 × 10^−96^) and bacterial (3.1-fold enrichment, *P*=2 × 10^−34^) diseases (Supplementary Data 10).

We note that many of the eQTL observed in neutrophils may also be observed in monocytes (Supplementary Fig. 5) reinforcing the need for additional follow-up to resolve complexity of how a variant may predispose to disease through effects in one or more cell types. We therefore explored two eQTL in greater detail to demonstrate how integrated analysis can provide novel insights into disease.

### An eQTL of PADI4 affects rheumatoid arthritis susceptibility

Rheumatoid arthritis is a systemic autoimmune disease afflicting ∼0.5–1% of adults in which autoimmune destruction of synovial joints occurs^53^. A major feature of disease is the presence of autoantibodies directed against citrullinated proteins. We found that 13 of 101 loci (a threefold enrichment compared with background, 95% CI 1.5–5.4,*P*=8 × 10^−4^) recently reported to be associated with rheumatoid arthritis risk in the largest meta-analysis to date^54^ are eQTL in neutrophils. These include rs2240335, an eQTL for *PADI4* that is in near-complete linkage disequilibrium with rs230188 (*r*^2^=0.93 in our data set). The A allele at rs2240335 is associated with elevated risk of rheumatoid arthritis and together with rs230188 is a genome-wide significant correlate of rheumatoid arthritis risk^54^. Intriguingly, rs2240335-A is associated with increased expression of *PADI4* in neutrophils but reduced expression in monocytes (Fig. 5c). rs74058715-T, a nearby SNP independent to rs2240335 (*r*^2^=0.02), is associated with reduced *PADI4* expression in both cell types providing an additional instrument to probe the role of *PADI4* in neutrophils in rheumatoid arthritis risk (Fig. 5b). Examination of histone modifications in the region show that rs2240335 lies in a region marked by histone 3 lysine 27 acetylation (H3K27Ac) and histone 3 lysine 4 monomethylation (H3K4me1) in neutrophils but not in monocytes whereas rs74058715 is in a region marked in both cell types (Fig. 5d), consistent with the cell type in which the eQTL is seen and suggesting rs2240335 as the functional variant as opposed to rs230188. Conditioning on rs2240335 does not reveal a secondary eQTL peak. Following from this prediction, rs74058715-T is nominally associated with reduced rheumatoid arthritis risk (*P*=0.05 in the largest meta-analysis of rheumatoid arthritis) showing directional consistency. We note that rs74058715 tags at least six other SNPs (linkage disequilibrium (LD)>0.8) that are nominally associated with rheumatoid arthritis, and therefore may not itself be the causal SNP. Subsequent to oxidative responses by stimuli including rheumatoid factor, PADI4 post-translationally citrullinates histones and other proteins initiating NETosis, a process shown to be central to rheumatoid arthritis pathogenesis^55^,^56^,^57^. Because PADI4 expression is confined to neutrophils and monocytes^58^, these data support a model in which rs230188 tags rs2240335-A, which alters PADI4 expression and rheumatoid arthritis risk likely through its actions in neutrophils.

### Functional basis of rs1981760 in leprosy and Crohn’s disease

**Figure 6:**
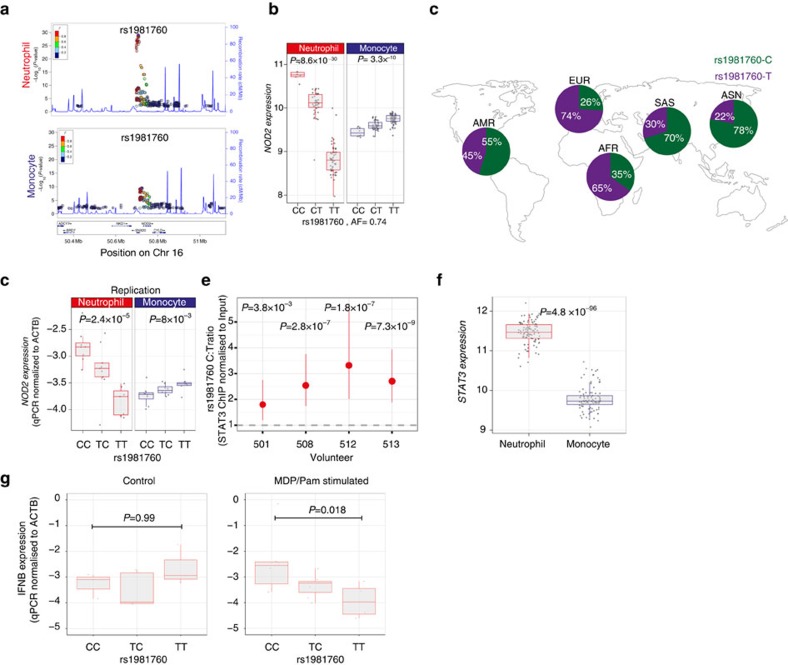
eQTL in NOD2 and their involvement in Leprosy and Crohn’s Disease(CD). (**a**) Regional association plots for rs1981760 and *NOD2* in Neutrophils (top) and monocytes (bottom). (**b**) The C (derived) allele at rs1981760, a variant located in an intergenic region between *NOD2* and *SNX20*, is associated with elevated *NOD2* expression in neutrophils and reduced *NOD2* expression in monocytes, *n*=101. (**c**) rs1981760 is remarkable for the profound differentiation this allele appears to have undergone in Asian populations with the derived C allele predominating in Asians (data from the 1000 genomes project^68^). rs1981760 and rs9302752 are in high linkage disequilibrium (*r*^2^=0.98 in our cohort). rs9302752-C is associated with leprosy risk in GWAS reports from Chinese populations^60^. The C variant has a mild protective effect in CD independent of the deleterious 3020insC frameshift mutation in *NOD2* (ref. 59). (**d**) Quantitative PCR of *NOD2* with two different primer sets performed blinded to genotype status in an independent set of 23 donors replicates the finding. Data representive of one primer-pair. (**e**) The rs1981760 SNP is located within a STAT3 transcription factor ChIP-seq binding site in MCF10A-Er-Src, an epithelial cell line (ENCODE). We investigated whether rs1981760 alters STAT3 binding in neutrophils by ChIP of STAT3 in four rs1981760 heterozygotes (screened to not have the 3020insC frameshift mutation in NOD2). Allele-specific probes in a digital droplet PCR assay demonstrate that the C allele is significantly more frequently immunoprecipitated with STAT3 than the T-allele. *P*-values denote a χ^2^-test comparing expected versus actual ratio of positive and negative droplets for each donor. Tails show 95% CI of the enrichment estimate. (**f**) *STAT3* expression levels differ between neutrophils and monocytes as measured by gene expression array, *n*=101. (**g**) After stimulation with muramyl dipeptide(MDP), the ligand for NOD2 and Pam3CSK4 (a co-stimulant) neutrophils from rs1981760-CC homozygous individuals express significantly higher levels of *IFNB* mRNA than TT homozygotes, and heterozygotes express intermediate levels (left panel). No such association is apparent in mock-treated control neutrophils (right panel). (**b**,**d**,**f**,**g**) Box lower and upper border denote 25th and 75th centiles, respectively, central line denotes median and whiskers extend to 1.5 × IQR.

Leprosy, a disease caused by the infectious agent *Mycobacterium leprae*, and CD, an autoimmune inflammatory bowel disease, show profound overlap in genetic architecture^59^. The ancestral T allele of rs1981760 is associated with increased susceptibility of leprosy, particularly multibacillary disease^60^ and yet has weak protective effects on CD, independent to the major CD-associated missense mutations^59^. We observed a strong association between rs1981760-T and reduced expression of both *NOD2* (*P*=8 × 10^−30^, variance explained=39%), and the adjacent gene *SNX20* (*P*=7.8 × 10^−10^) in neutrophils and conversely elevated expression in monocytes (*P*=3.3 × 10^−10^) (Fig. 6a,b). The frequency of rs1981760-C is strikingly differentiated in Asians compared with other populations (78% in Asians versus 26% in Europeans from the 1000G project; Fig. 6c). To further test the mechanism of this finding and functional consequences, we enrolled an independent cohort of 23 individuals. We were able to replicate the observed eQTL by quantitative PCR with two independent probe sets (Fig. 6d). The rs1981760 polymorphism is reported to alter *STAT3* binding in some ENCODE cell lines. We note that rs1981760 is in complete LD with rs9302752, the SNP first reported to be associated with leprosy and CD, but since rs1981760 is the primary eQTL signal, and in view of it potentially modifying transcription factor binding, we pursued rs1981760. Conditioning on rs1981760 does not reveal a secondary eQTL peak. To test whether the polymorphism alters STAT3 binding in neutrophils we performed ChIP with an antibody directed against STAT3 in four heterozygotes. Using allele-specific probes in a digital droplet PCR reaction designed to accurately quantify the number of each allele, we observed significantly fewer copies of immunoprecipitated DNA containing T alleles than C alleles after ChIP in each individual (Fig. 6e, Supplementary Data 11) consistent with the hypothesis that the T allele reduces STAT3 binding either alone or potentially in a complex with other transcription factors. Moreover, *STAT3* is markedly differentially expressed in neutrophils and monocytes (Fig. 6f). Finally, we stimulated neutrophils from individuals with the NOD2 ligand muramyl dipeptide (supplemented with Pam3-CSK4 as a synergistic agonist). Individuals with the CC genotype expressed significantly greater levels of mRNA for *IFNB* (*P*=0.02; Fig. 6g) after stimulation. These data demonstrate the rs1981760 affects *NOD2* expression and subsequent interferon β (IFNB) responses to its ligand. Notably, eQTL in neutrophils are enriched for genes in the IFNB network (*P*=5.9 × 10^−4^). Therefore these data suggest that type-1 interferons and neutrophils may be involved in leprosy as has been shown in tuberculosis^61^.

### Neutrophil eQTL are enriched in regions of natural selection

Neutrophils are an evolutionarily ancient cell. In light of the rs1981760 observation, we systematically examined whether regulatory variants in neutrophils overlap regions that are identified as being subject to selection in systematic human surveys^62^. Indeed, 1,527 variants that are eQTL for a total of 39 genes lie in regions that have been subject to selection in humans (Supplementary Data 12), a 2.4-fold (95% CI 2.33–2.6) enrichment (*P*=1.04 × 10^−195^) relative to the 20,464 variants tested in our study that lie in annotated regions of selection. Many of the genes showing eQTL driven by variants in such regions are known to be involved in leukocyte biology; for example, the chemokine receptors *CCR1* (ref. 63) and *CCR3*, or *GUSB*, a gene in which mutations cause mucopolysaccharidosis VII (ref. 64) in which recurrent respiratory infections are common. Others, such as *HERC2* involved in eye colour^65^ and for which the second strongest eQTL in neutrophils is observed, may suggest new hypotheses for operative selective forces and the cells to yield the patterns observed. Therefore, regulatory variants in neutrophils are likely to have been subject to selection, reinforcing their biological relevance.

In conclusion, these data illustrate the utility of eQTL mapping in different leukocyte subsets and integration with epigenetic and disease-association data to reveal mechanisms of disease.

## Methods

### Volunteer enrolment and cell purification

This study was approved by the Oxfordshire Research Ethics Committee (COREC reference 06/Q1605/55) and each individual gave informed consent to participation. The median age of the 101 included participants was 31 years (IQR 24–41), 50 were male and 51 female. For this substudy of our previously reported work^19^ we recruited 144 healthy Caucasian volunteers in Oxford, United Kingdom through the Oxford Biobank. A trained professional nurse and/or a medical doctor conducted a verbal review of clinical history to determine eligibility based on the absence of major chronic illness, current medication administration or symptoms of infection. We excluded 43 individuals because we did not have genotyping information at the time of this report (23 individuals) or the sample was an outlier after principal components analysis (20 individuals). We used an automated outlier detection algorithm ‘detectOutlier’ (*lumi* package) based on Euclidean distance to the centre of the cluster with iterative outlier removal and normalization. Whole blood was collected into sodium–heparin containing blood collection tubes (Becton Dickinson) and processed with 4–6 h after collection.

We isolated peripheral blood mononuclear cells by density-gradient centrifugation of blood-diluted with Hanks Buffered Saline solution (HBSS, Life Technologies, UK) layered on Lymphoprep (Axis-Shield, Norway) and sorted CD14+ monocytes using magnetic-activated cell sorting (MACS, Miltenyi Biotech) according to the manufacturer’s instructions.

We isolated granulocytes using density-gradient centrifugation in which blood was layered on Polymorphrpep (Axis-Shield, Norway) according to the manufacturer’s instructions. To remove red blood cell contamination we exposed granulocytes to endotoxin free cell-culture water (Life Technologies, UK) for 30 s and restored isosmosis using 2X HBSS. As neutrophils and eosinophils share density properties (1.085 g ml^−1^), and previous studies demonstrated that *ex vivo* granulocyte transcriptomes are dominated by eosinophil transcripts despite their relative paucity^27^ (<5% of granulocytes), we further isolated CD16+ granulocytes as a pure population of neutrophils using CD16+ microbeads. Purity assessed on a representative sample was >90%. We performed full-blood counts on a Sysmex haematology analyser in a certified clinical laboratory; median neutrophil counts were 3.07 (IQR 2.52–3.78) × 10^9^ cells l^−1^. We note that the use of beads directed against CD16 may, in principle, alter or stimulate neutrophils although all purification steps were carried out on ice or at 4 °C. Cell viability after isolation was >80%. Lysed cell pellets were immediately cryopreserved until extraction.

### Genotyping

A total of 733,202 variants were genotyped using the Illumina HumanOmniExpress-12v1.0 Beadchip. QC steps included removal of individuals that were outliers by PCA, had poor genotyping call rates or heterozygosity measures. Variant QC included removal of SNP’s with MAF<5%, HWE<1 × 10^−6^ or poor call rates. Genome-wide imputation was performed by prephasing a scaffold of 588,170 SNP’s with SHAPEIT^66^ and imputation in IMPUTE2^67^ using the 1000G phase 1 release as the reference. Imputed SNP or indels with an info score <0.9, MAF<5% or departure from HWE (1 × 10^−3^) were removed. In total 5,680,354 variants were included in the eQTL analysis. Locations, where reported, are according to the human genome build GRCh37. Measures of LD, where mentioned, are based on 1000G CEU^68^ or, if specifically mentioned, from this cohort of volunteers.

### Gene expression analysis

During optimization of methods to isolate RNA from neutrophils we found that commonly used column-based isolation techniques were vastly outperformed by phenol–chloroform extraction using Trizol LS (Life Technologies) according to the manufacturer’s instructions. This may be because a higher concentration of chaotropic and reducing agent is required to fully inhibit the abundance of nucleases in neutrophils. Therefore RNA was extracted using Trizol LS and further cleaned using the RNA MinElute cleanup kit (Qiagen, UK). RNA was quantified and integrity assessed using a Bioanalyser RNA 6000 Nano kit (Agilent, UK). Gene expression was quantified using the Illumina HumanHT-12 v4 BeadChip gene expression array platform with 47,231 probes according to the manufacturer’s instructions. Samples were randomized across expression chips and run in a single batch.

Gene expression data were normalized using random-spline normalization, transformed by variance-stabilizing transformation and sample outliers were iteratively removed and normalization repeated. We note that of the 123 samples hybridized, 1 failed and 20 were removed as they were sample outliers-almost universally (19/20 samples) due to cRNA quality with these individuals having RNA size distribution<800 by BioAnalyser RNA 6000 Nano kit evaluation. We therefore did not obtain replacement arrays and hence all the data in this study derive from a single batch. Probe sequences mapping to more than one genomic location or regions with underlying polymorphisms frequent in >1% of the population were excluded from eQTL analysis (*n*=1,8220 probes). Only probes that were expressed and detected (GenomeStudio probe detection *P*<0.01) in neutrophils (*n*=11,023 probes for 9,147 genes) were included in the primary analysis. For comparisons between neutrophils and monocytes, 10,012 probes for 8362 genes were included from 99 individuals.

### eQTL mapping

We tested for association between genetic variation (using dosage estimates for imputed variants) within 1 Mb of the expression probe (*cis*) or more distantly (*trans*), and gene expression in a linear regression framework implemented in ‘MatrixEQTL’^35^ in *R* (Matrix_eQTL_main function), incorporating principal components as covariates to account for hidden confounders (empirically determined number of PCs=15). We filtered results by a false discovery rate of 5% for *cis* associations or 1% for *trans* associations. We caution that this study, due to its modest size, is not adequately powered to exhaustively identify eQTL in neutrophils, or to map *trans* eQTL.

### Annotation of eQTL with genomic features

Identified eQTL were annotated by location relative to the transcription start site or gene structures using data from the UCSC genome browser (GRCh37/b19)^69^.

We accessed whole-genome bisulfite sequencing and histone ChIP data generated by the BLUEPRINT consortium in primary human monocytes and neutrophils from 4 to 8 individuals (depending on feature). We defined features marked by an epigenetic mark as those present in at least half of all individuals (Supplementary Table 5).

For analyses of eQTL overlap with the location of epigenetic marks^46^, enhancers^47^,^48^, transcription factors^49^, conserved microRNA binding sites^70^ and in regions that have been under selection^62^, the location of the given feature relative to an eQTL was calculated using the ‘GenomicRanges’ package in R with the ‘distanceToNearest’ function. Fisher’s exact test was used to evaluate enrichment relative to all imputed variants tested for eQTL (that is, those within 1 Mb of a gene expressed in neutrophils and monocytes). We note that an implicit assumption in the Fisher’s test is of independence of loci. The code used to generate the results is available from the authors.

### Overlap between eQTL and trait-/disease-associated variants

All traits listed in the NHGRI Catalog GWAS (1138 traits as at 14 October 2014) were abstracted and expertly curated into one or more categories according to the disease system affected (28 potential categories) blind to GWAS or eSNP data. Groups were defined based on organ specificity of a particular trait, disease process or type. These included cardiovascular, respiratory, gastroenterological, urological, rheumatological, neurological, renal, endocrine, haematological, dermatological, bone, cancer, immunity and inflammation, autoimmune, allergy, genetic, viral infection, bacterial infection, parasitic disease, measurement, physiological, metabolic, chronic or degenerative disease, reproduction, drug related. Classification of a given trait was possible into multiple groups and to capture this diversity, for each trait we assigned trait membership into up to three possible groups.

A Fisher’s exact test comparing the proportion of eQTL that are trait-associated variants or in linkage disequilibrium with these variants (*r*^2^>0.8 in 1000G Caucasian populations) with the proportion of all tested variants that are associated variants was performed stratified by disease category.

### Neutrophil stimulation

To identify the role of rs1981760 in NOD2 downstream effects, we stimulated 5–10 × 10^6^ freshly isolated neutrophils resuspended at 2 × 10^6^ cells per ml in RPMI1640 supplemented with 10% foetal calf serum and L-glutamine with 1 μg ml^−1^ muramyl dipeptide (n-acetyl) and 1 μg/ml Pam3CSK4, (both from Invivogen, UK) for two hours. Primer sequences for quantitative PCR are reported in Supplementary Data 13.

### Chromatin immunoprecipitation

Crosslinked DNA from neutrophils was stored and subjected to ChIP after fragmentation of crosslinked DNA by sonication consisting two sets of nine cycles of 30 s each in a BioRuptor (Diagenode, Belgium) at high power. A mouse anti-human STAT3 antibody (Cell Signaling-NEB, UK, catalogue #4904S) was used at a 1:50 dilution in conjunction with magnetic beads to immunoprecipitate STAT3 bound DNA. Quality control of ChIP-ped DNA was achieved by quantification on a Qubit 2.0 fluorometer (Invitrogen) using the Quant-iT dsDNA HS Assay Kit (Invitrogen).

### Assessment of allele-specific STAT3 binding in neutrophils

For allele-specific digital droplet PCR quantification of rs1981760-C compared with rs1981760-T we designed allele-specific probes (Supplementary Data 13), and performed amplification using the digital droplet PCR Supermix for Probes (BioRad) according to the supplied protocol. The probe for the T and C allele were designed to fluoresce in different channels allowing simultaneous detection of both in a single reaction. Droplets were generated on a q × 100 droplet generator (BioRad, UK) and droplets read on a q × 100 droplet reader. Probes targeting SOCS3 and JAK3 were used as positive controls for ChIP of STAT3, and RAB4 as a negative control. Input DNA as well as DNA obtained after ChIP was amplified on the same plate. For allele-specific wells, we performed technical duplicates.

The proportion of positive droplets relative to negative droplets is associated with the absolute concentration of the product according to a Poisson distribution. We therefore exploited this to empirically calculate the expected proportion of C versus T droplets (so as to take into account possible probe efficiency differences) and compared this to the actual ratio after ChIP. *P*-values using a *χ*^2^-test with two degrees of freedom were used to estimate the probability of the observed versus the expected ratio being due to chance.

### Software used for analysis

Analyses were conducted in *R*, using the following packages: limma; lumi; ‘annotate’; ‘vsn’, ‘GenomicRanges’, ‘qvalue’, ‘data.table’. For data presentation the ‘gridExtra’, ‘ggbio’, ‘ggplot2’ and ‘VennDiagram’ packages were used. Local association plots were created using LocusZoom. Ingenuity Pathway Analysis where used, was performed using a background set of all human genes on the Ilumina array. A listing of external files and their sources is given in Supplementary Data 14. FDR where shown denotes the false discovery rate calculated using the Benjamini Hochberg procedure using the p.adjust function.

## Additional information

**How to cite this article:** Naranbhai, V. *et al*. Genomic modulators of gene expression in human neutrophils. *Nat. Commun.* 6:7545 doi: 10.1038/ncomms8545 (2015).

## Supplementary Material

Supplementary Figures Supplementary Figures 1-5

Supplementary Data 1 Top *cis* eQTL per probe in neutrophils ranked by degree of statistical confidence.

Supplementary Data 2 Association between copy-number variants and gene expression.

Supplementary Data 3 *trans* eQTL in neutrophils ranked by statistical confidence.

Supplementary Data 4 eQTL in genes involved in neutrophil function categorised by role in neutrophil function and relationship, if any, with Mendelian disorder.

Supplementary Data 5 Summary of epigenetic features in neutrophils and monocytes from the BLUEPRINT project.

Supplementary Data 6 Enrichment of eQTL in regions marked by histone modifications, DNA methylation, transcription factor binding, microRNA binding or transcribed enhancers.

Supplementary Data 7 Differential expression of transcription factors in neutrophils and monocytes ranked by log fold change in expression.

Supplementary Data 8 eQTL in neutrophils that are in regions bound by conserved microRNA.

Supplementary Data 9 Enrichment of eQTL in neutrophils that are in LD (r^2^ >0.8) with or are variants associated with a complex trait listed in the NHGRI GWAS catalog according to disease category.

Supplementary Data 10 eQTL in neutrophils that are variants, or are in LD (r^2^ >0.8) with variants associated with a complex trait listed in the NHGRI GWAS catalog according to disease category

Supplementary Data 11 Neutrophil eQTL in regions of the genome identified as being subject to selection in Grossman *et al*.

Supplementary Data 12 Results of droplet digital PCR for the ratio of rs1981760-C:rs1981760-T after chromatin immunoprecipitation of STAT3 from crosslinked neutrophil DNA from rs1981760 heterozygote individuals.

Supplementary Data 13 Primer and probe sequences for oligonucleotides used in this study.

Supplementary Data 14 Listing of additional data files and source for external files to which reference is made in this study.
