# Supplementary material for: Genomic modulators of gene expression in human neutrophils
Source: Nat Commun. 2015 Jul 7;6:7545. doi: 10.1038/ncomms8545 (PMC4507005; doi:10.1038/ncomms8545)
Supplement: Supplementary Figures — 1-5 [file ncomms8545-s1.pdf]

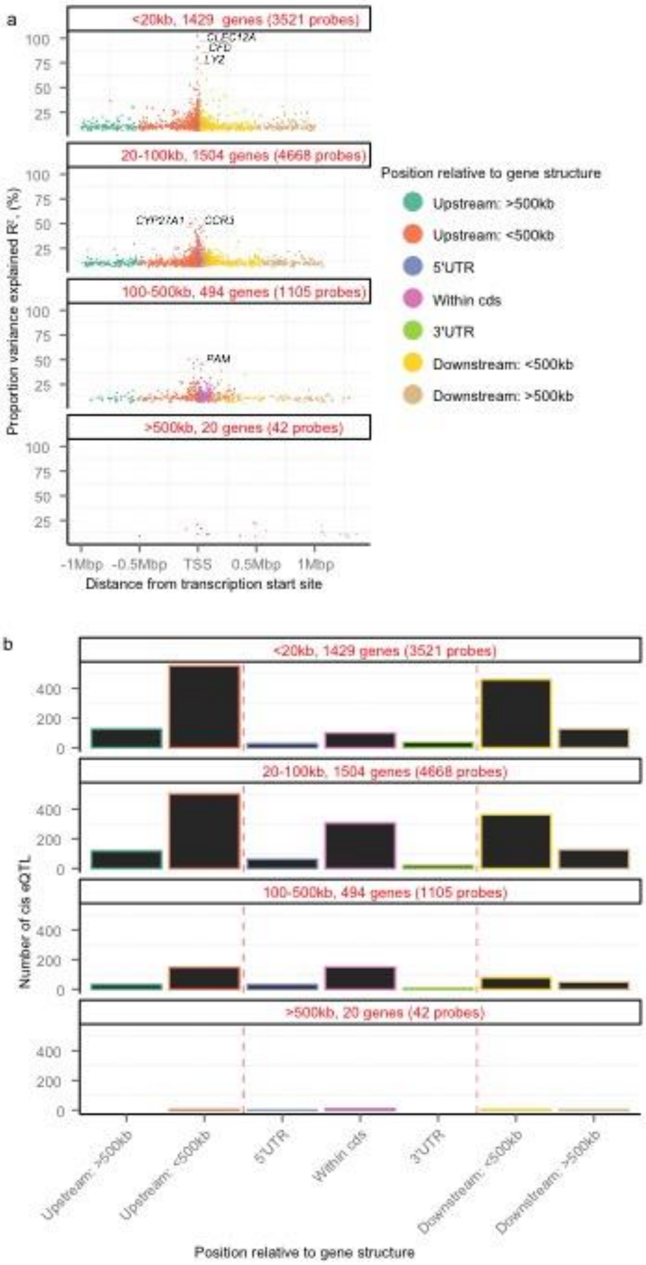

Supplementary Figure 1. Effect size and location of *cis* eQTL in neutrophils relative to transcription start site (TSS) (a) or gene structure (b), partitioned by gene size. Each point in (a) denotes the peak eQTL for a gene and is plotted according to the proportion of variance explained by the variant with strongest statistical association with gene expression and location of the variant relative to the TSS. Several genes with large effect sizes are annotated. (b) shows the number of *cis* eQTL identified in neutrophils according to the location of the peak eQTL relative to gene structures.

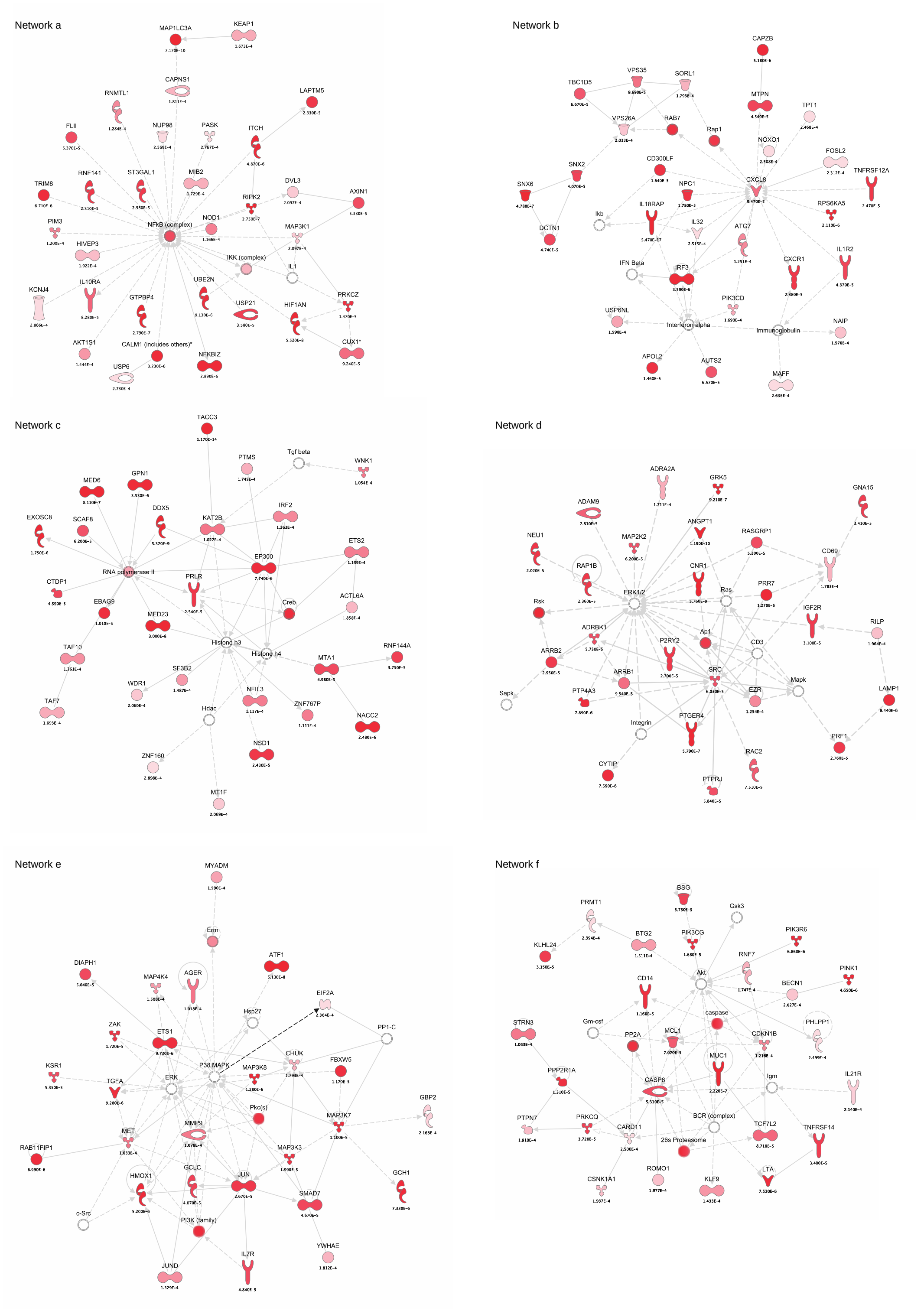

Network a
MAP1LC3A
KEAP1
CAPNS1
RNMTL1
LAPTM5
FLII
NUP98
PASK
ITCH
TRIM8
RNF141
ST3GAL1
MIB2
DVL3
AXIN1
RIPK2
NOD1
NFkB (complex)
PIM3
HIVEP3
MAP3K1
IKK (complex)
IL1
IL10RA
UBE2N
KCNJ4
GTPBP4
USP21
HIF1AN
PRKCZ
AKT1S1
CALM1 (includes others)*
NFKBIZ
CUX1*
USP6
Network b
CAPZB
VPS35
SORL1
TBC1D5
MTPN
TPT1
VPS26A
RAB7
Rap1
NOXO1
SNX2
CD300LF
FOSL2
TNFRSF12A
SNX6
NPC1
CXCL8
RPS6KA5
Ikb
IL18RAP
IL32
DCTN1
ATG7
IL1R2
IFN Beta
IRF3
CXCR1
USP6NL
PIK3CD
Interferon alpha
Immunoglobulin
NAIP
APOL2
AUTS2
MAFF
Network c
TACC3
PTMS
Tgf beta
WNK1
MED6
GPN1
IRF2
EXOSC8
SCAF8
DDX5
KAT2B
ETS2
EP300
RNA polymerase II
CTDP1
PRLR
ACTL6A
EBAG9
Creb
MED23
TAF10
Histone h3
Histone h4
RNF144A
SF3B2
MTA1
WDR1
NFIL3
ZNF767P
Hdac
NACC2
TAF7
NSD1
ZNF160
MT1F
Network d
ADRA2A
GRK5
GNA15
ADAM9
ANGPT1
NEU1
MAP2K2
RASGRP1
CD69
RAP1B
CNR1
PRR7
ERK1/2
Ras
Rsk
IGF2R
ADRBK1
Ap1
RILP
P2RY2
CD3
ARRB2
SRC
ARRB1
Mapk
Sapk
PTP4A3
EZR
LAMP1
Integrin
PTGER4
PRF1
CYTIP
RAC2
PTPRJ
Network e
MYADM
Ezrn
ATF1
DIAPH1
MAP4K4
AGER
EIF2A
Hsp27
PP1-C
ZAK
ETS1
CHUK
KSR1
P38 MAPK
MAP3K8
FBXW5
TGFA
ERK
Pkc(s)
GBP2
MMP9
MAP3K7
MET
RAB11FIP1
MAP3K3
GCLC
GCH1
HMOX1
JUN
SMAD7
c-Src
PI3K (family)
YWHAE
JUND
IL7R
Network f
BSG
PRMT1
Gsk3
BTG2
PIK3CG
PIK3R6
KLHL24
RNF7
Akt
PINK1
CD14
BECN1
STRN3
Gm-csf
caspase
PHLPP1
PP2A
MCL1
CDKN1B
MUC1
PPP2R1A
IL21R
CASP8
Igm
BCR (complex)
PTPN7
PRKCQ
CARD11
TCF7L2
TNFRSF14
26s Proteasome
ROMO1
CSNK1A1
KLF9
LTA

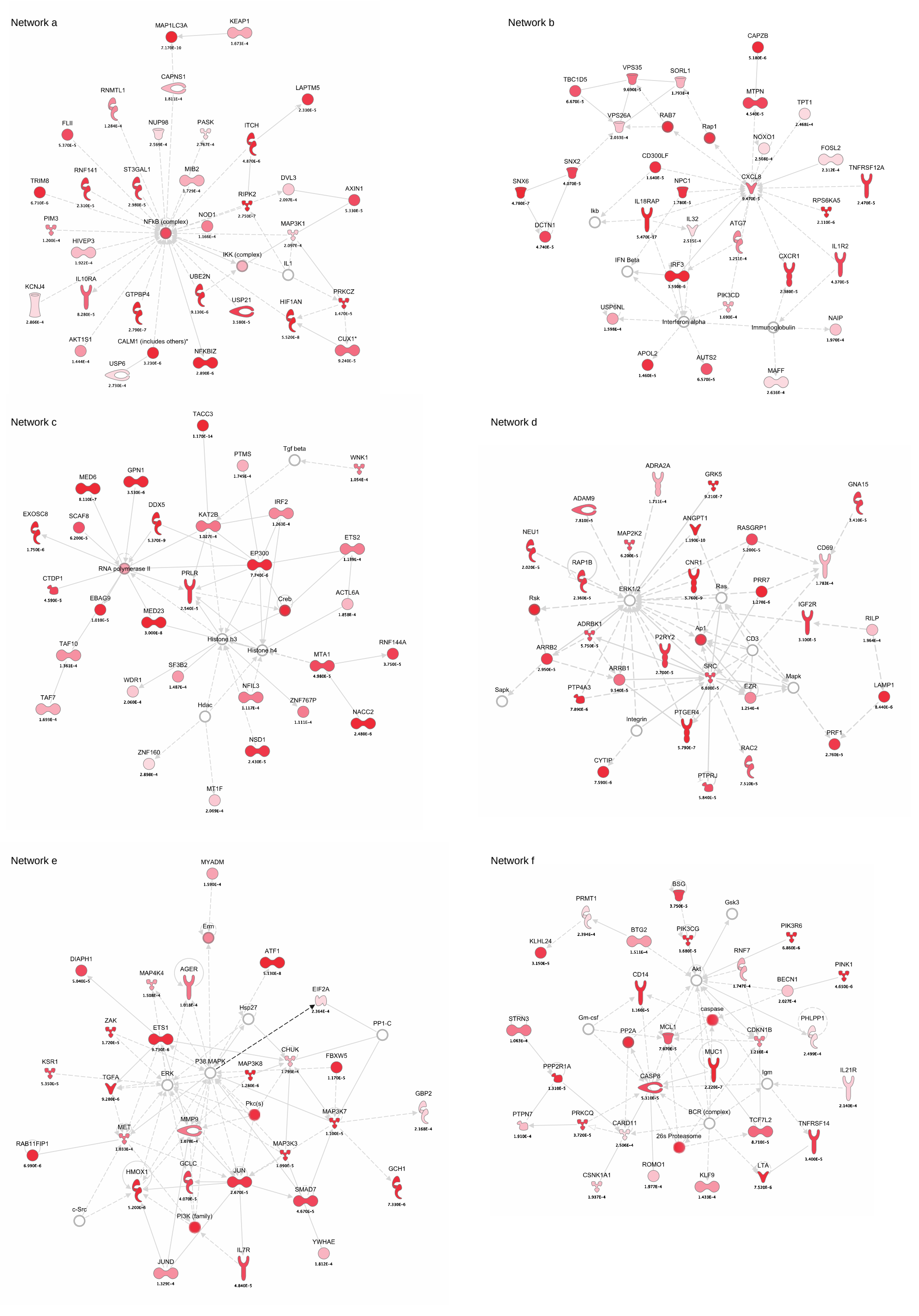

Supplementary Figure 2. Pathway analysis of genes with neutrophil specific *cis* eQTL. Amongst 975 genes with an eQTL in neutrophils only, a number of gene networks are identified as enriched for the genes showing *cis* eQTL specific to neutrophils (top 10 shown here with intensity of red shading related to p value of eQTL, gene names and p values shown above and below

molecular symbol). Network a ($p=1x10^{-36}$, Fishers exact test) involves genes associated with NFkB such as *MAP1LC3A* encoding LC3 involed in autophagy ($p=7.1x10^{-10}$), *TRIM8* ($p= 6.7x10^{-6}$) which modulates p53 activity and *CALM1* (calmodulin 1) ($p=3.2x10^{-6}$); network b ($p=1x10^{-30}$) involving *CXCL8* (IL8) ($p= 9.5x10^{-5}$) a critical modulator of neutrophil recruitment and behaviour with associated genes showing eQTL including *IRF3* ($p=3.6x10^{-6}$), *IL18RAP* ($p=5.5x10^{-17}$) and *CXCR1* (IL8 receptor) ($p=2.4x10^{-5}$); network c ($p=1x10^{-30}$) centred on histones H3 and H4; network d ($p=1x10^{-26}$) involving genes linked with ERK1/2 MAP kinases; network e ($p=1x10^{-26}$) also focused on ERK and P38 MAPK and involving a network of genes including many transcription factors such as *ETS1* ($p=9.7x10^{-6}$), *ATF1* ($p=5.1x10^{-8}$) and *JUN* ($p=2.7x10^{-5}$); network f ($p=1x10^{-26}$) involving Akt and caspases; network g ($p=1x10^{-26}$) including eQTL involving *ATM* ($p=1.5x10^{-18}$), heat shock proteins *HSPA1A* and *HSPA1B* ($p=1x10^{-6}$) and *MCM5* ($p=3.4x10^{-11}$); network h ($p=1x10^{-23}$), network i ($p=1x10^{-19}$) involving genes linked to the nuclear transcriptional regulator NURP1 (also known as p8) showing eQTL such as the phopsholipid gene *PCTP* ($p=9.5x10^{-13}$); and network j ($p=1x10^{-18}$) involving IL12, PI3K and TCR which includes genes showing *cis*-eQTL such as *STAT5A* ($p=1.9x10^{-5}$) important in GM-CSF signaling and control of granulocyte homeostasis[69] and IDO1 ($p=4.8x10^{-6}$) which has a role in neutrophil self-regulation through tryptophan catabolism[70].

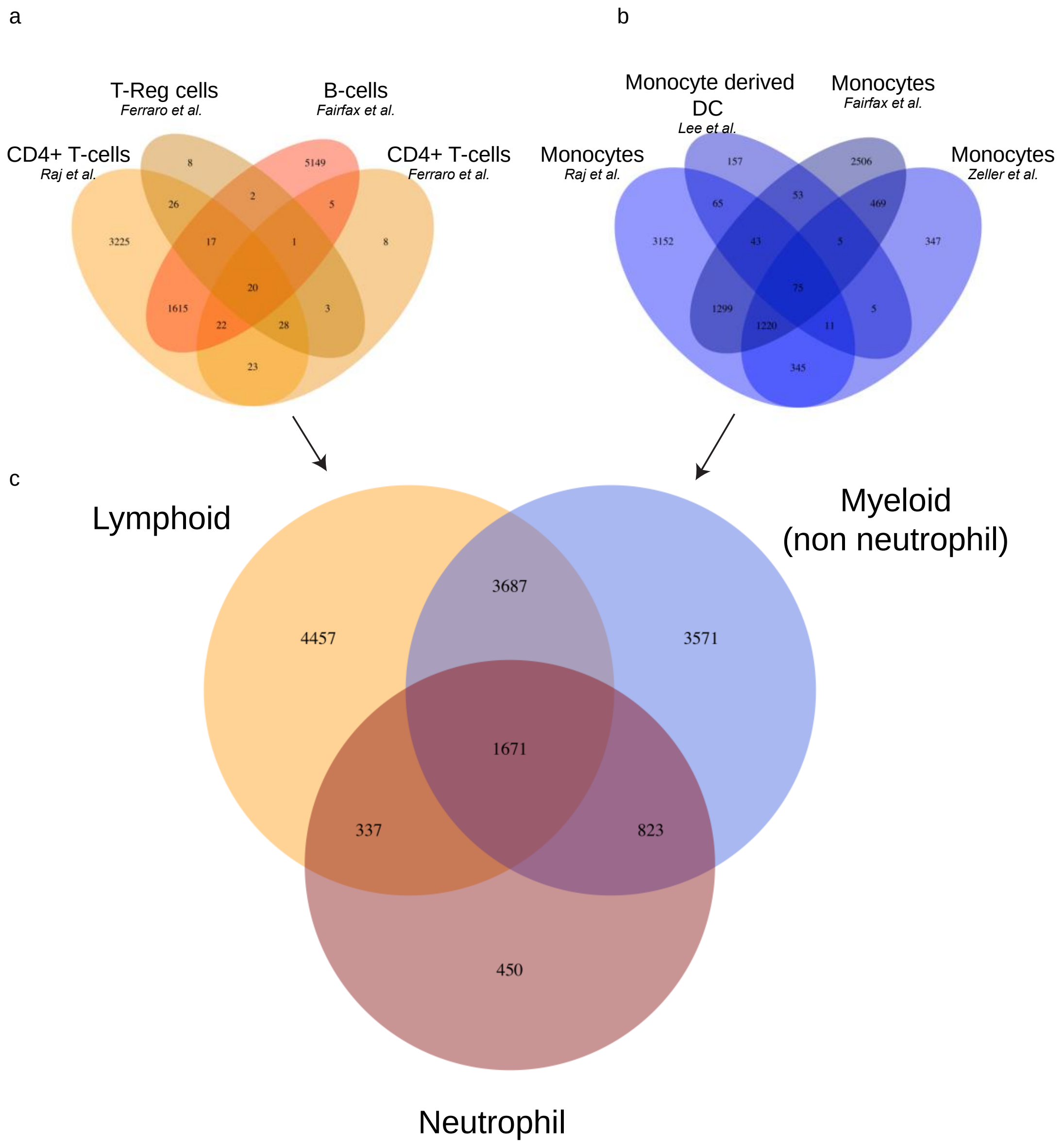

Supplementary Figure 3. Euler diagrams showing overlap of genes with an eQTL previously observed in lymphoid (a) or myeloid (b) cell types and their overlap with genes with eQTL in neutrophils (c). We extracted lists of genes reported to have a significant eQTL from several previous studies and show the number of overlapping or non-overlapping genes for the four studies reporting eQTL in lymphoid cells[14,15,17] (a) or myeloid cells[13-16] (b). Overlapping the lists of genes from A and B, with the list of 3281 genes with an eQTL in neutrophils yields panel with number of overlapping genes noted (c).

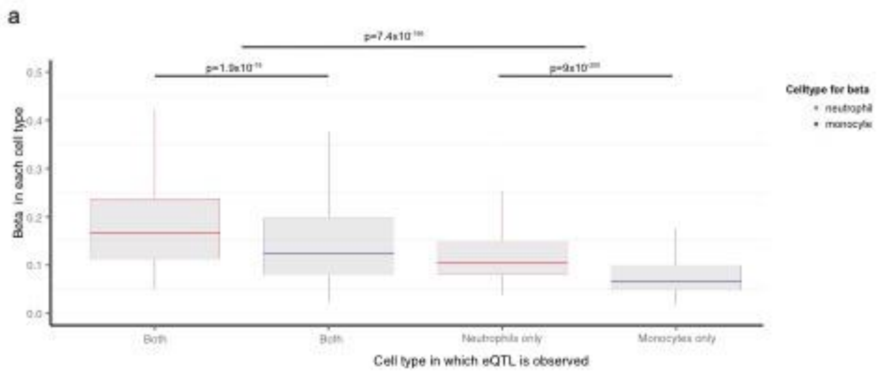

Supplementary Figure 4: Effect sizes of eQTL. These tend to be larger for eQTL that are observed in both cell types (labelled as both) or those seen in one cell type only and are larger in neutrophils than monocytes regardless of whether the eQTL is observed in both cell types or one only. Box lower and upper border denote 25th and 75th centiles respectively, central line denotes median and whiskers extend to 1.5*IQR

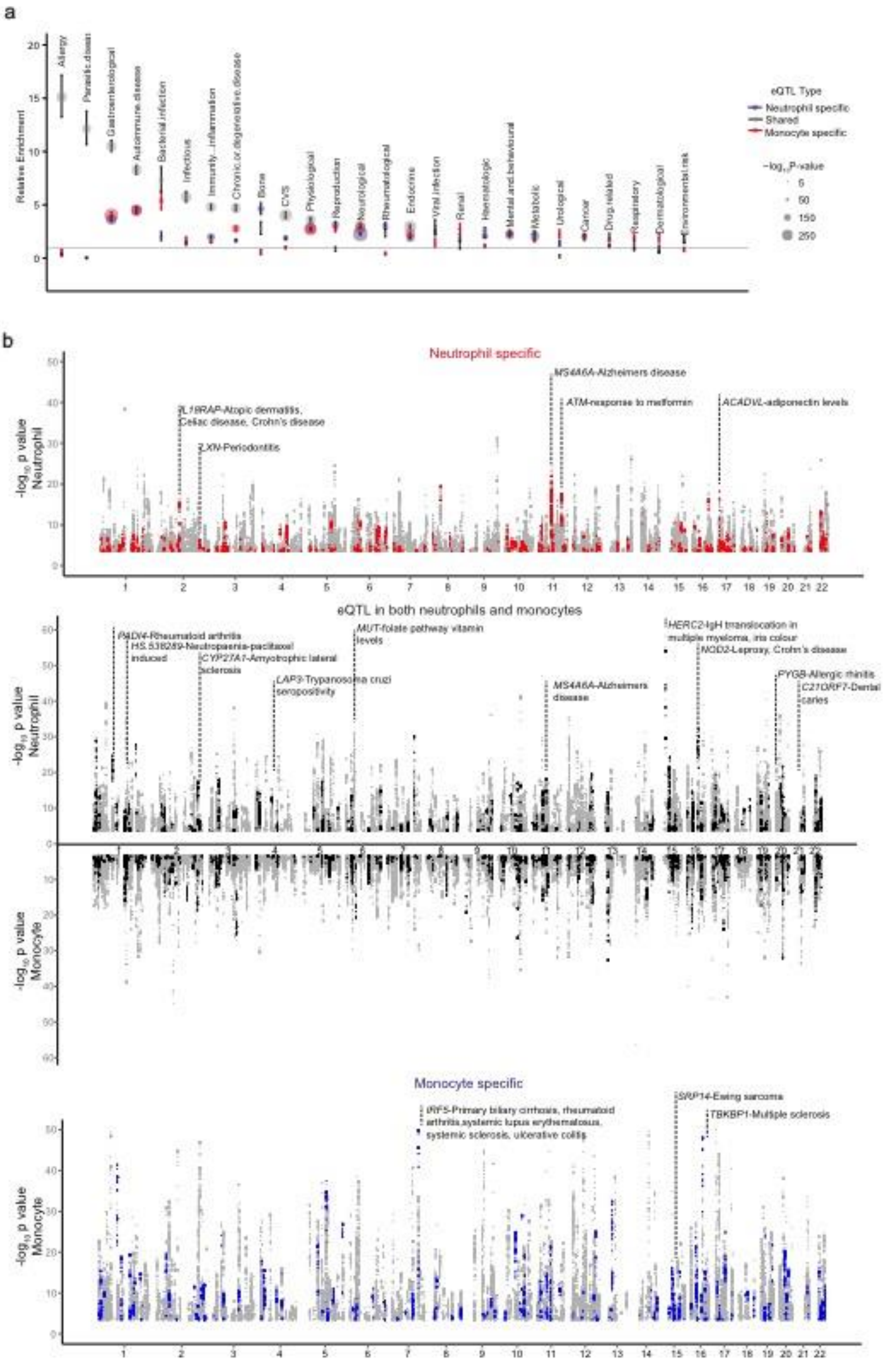

Supplementary Figure 5: Shared and cell type specific *cis* eQTL are enriched for variants associated with diseases or traits in GWAS studies. (a) Enrichment of shared and cell type specific eQTL for variants reported in the GWAS catalog as associated with a trait or disease by GWAS ontology category. For each category we compared the proportion of eQTL

variants associated with a trait in the relevant category to the proportion of all variants tested that had this property by a Fisher’s exact test. Tails show 95% CI of the enrichment estimate (b) Manhattan plots demonstrating significant (all FDR<0.05) shared (black), neutrophil-specific (red) or monocyte-specific (blue) eQTL that are either associated with a trait in the GWAS catalog (color) or not (grey). An arbitrary selection of diseases for which the risk variant (or a variant in linkage disequilibrium r^2>0.8) is also an eQTL are highlighted.
